# Anti-inflammatory Mechanism of Ruzu Bitters on Diet-Induced Nonalcoholic Fatty Liver Disease in Male Wistar Rats

**DOI:** 10.1155/2020/5246725

**Published:** 2020-07-23

**Authors:** Olubanke O. Ogunlana, Oluseyi E. Ogunlana, Tobi S. Adekunbi, Babatunde O. Adetuyi, Bose E. Adegboye, Franklyn N. Iheagwam

**Affiliations:** ^1^Department of Biochemistry, College of Science and Technology, Covenant University, Ota, Ogun State, Nigeria; ^2^Department of Biological Sciences, Crawford University, Igbesa, Ogun State, Nigeria; ^3^Department of Natural Sciences, Precious Cornerstone University, Ibadan, Oyo State, Nigeria

## Abstract

Nonalcoholic fatty liver disease (NAFLD) has become notorious globally. Increasingly emerging evidence shows that NAFLD is strongly associated with inflammation, with proinflammatory cytokines such as interleukin-2 (IL-2), interleukin-6 (IL-6), and tumour necrosis factor*-α* (TNF-*α*) playing a vital role in its progression. In this work, an attempt was made to verify the anti-inflammatory activity of Ruzu herbal bitters (RHB), an antiobesity medicinal concoction, on NAFLD induced by a high-fat diet (HFD) in albino Wistar rats. Twenty-five (25) rats were divided into five groups as follows: Group 1, the normal control, was maintained on standard rat chow and received normal saline (1 ml/kg body weight (BW)/day) for twelve weeks. The other groups were maintained on HFD for twelve weeks. Thereafter, groups 2–5 were treated with pioglitazone (4 mg/kg BW/day), RHB (0.6 ml/kg BW/day), normal saline (1 ml/kg BW/day), and fenofibrate (10 mg/kg BW/day), respectively. The animals were sacrificed after the experimental period. Biochemical indicators of oxidative stress and inflammation were assayed in the liver according to standard methods. The histological features of the liver were also compared to assess liver damage. RHB significantly (*p* < 0.05) reduced body weight and liver index, inhibited oxidative stress, boosted antioxidant enzymes by increasing the activity and level of SOD and GSH, reduced proinflammatory markers (IL-2, IL-6, TNF-*α*), and reversed histological alterations induced by NAFLD in rat liver. In conclusion, the anti-inflammatory activity of RHB in the prevention of NAFLD in rats has been confirmed.

## 1. Introduction

Most acute and chronic liver disorders, comprising nonalcoholic fatty liver disease (NAFLD), are associated with inflammation. NAFLD, a chronic liver disease (LD), is one of the significant causes of LD in childhood, adolescence, and adulthood [1, 2]. NAFLD is a fatty LD, not caused by excess intake of alcohol, but linked with obesity and IR (insulin resistance) [3–5]. The occurrence of hepatic steatosis (HS) ≥5%, in the absence of other opposing LD aetiologies, such as use of medications, autoimmune hepatitis, hemochromatosis, Wilson's disease, chronic viral hepatitis, and/or significant alcohol consumption [6], is the signature of NAFLD. As an independent risk factor for cardiovascular diseases (CVD), NAFLD may be handy in predicting metabolic disorders, independent of other risk factors [7–9]. Obesity, diabetes, and metabolic syndrome (MS) have direct correlation with occurrence of NAFLD [10]. It must be noted that the major cause of morbidity and mortality in NAFLD is cardiovascular disorders. Affecting up to 35% of the population in many western nations, NAFLD is considered the most common LD [11]. Patients with simple steatosis, about 1–5%, would eventually develop actual cirrhosis, while 10–15%, with NASH (nonalcoholic steatohepatitis), a form of NAFLD, would eventually end up with complications leading to cirrhosis and hepatocellular carcinoma [12].

Viral hepatitis-induced and alcoholic LD were considered the leading causes of LD resulting in morbidity and mortality in developed countries for many years. Prevalence rates of 8.7% in Nigeria, 10–30% in the United States of America, 23% in Europe, 31% in South America, 32% in the Middle East, and 27% in Asia have been reported [13]. NAFLD prevalence in Nigerian diabetics is 9.5–16.7% and in nondiabetics is 1.2–4.5% [14, 15].

High dietary carbohydrate, synonymous with the pathogenesis of NAFLD, is an important risk factor of the disease. A diet rich in fructose, up to 60% of the daily energy requirement, may precipitate an increased liver lipid build-up resulting in accompanying IR [15]. The progression of NAFLD is helped by a high rate of consumption of saturated fatty acids and refined carbohydrates [16]. The pathogenesis of NAFLD has not been fully understood. A theory of a two-hit mechanism on the liver involving first excessive fat accumulation in the hepatocytes accompanied by IR has been proposed [2, 17] leading to multiple hepatic injuries and consequent inflammation, fibrosis, and oxidative stress. Recent studies on mice revealed that the risk and progression of NAFLD are increased by a HFD (high-fat diet) emanating from antioxidant imbalance [18].

Mediators of immunity, especially proinflammatory cytokines, have been reported to control many vital features of LD. Most tissues including the liver have minimal or no constitutive production of cytokines under physiological conditions [9]. The regeneration of liver tissues is mediated by cytokines [5]. The proinflammatory interleukin type cytokines and tumour necrosis factor are chief among the cytokines known to be key markers of fatty LD [13]. In the liver, TNF-*α*, a direct secretion by hepatocytes and Kupffer cells or indirect secretion by intestinal fat [14], is a main factor in the progression of NAFLD in both humans and animals. TNF-*α* inhibition in an animal model of NAFLD presents a promising therapeutic strategy [19]. The potential involvement of TNF-*α* in NAFLD pathophysiology was recently suggested by genetic studies on its polymorphisms [20].

Interleukins (IL-2 and IL-6), having a wide range of biological functions, stimulate several cells, such as immune cells [21, 22]. Although IL-6, initially considered as a hepatoprotective substance in liver steatosis, is capable of decreasing oxidative stress and averting mitochondrial dysfunction, it could also advance hepatic regeneration and repair, induce inflammation, sensitize the liver to injury, induce IR, stimulate hepatocyte apoptosis, and participate in NASH development [19]. Hepatic steatosis is enhanced by IL-6 pathway neutralization with tocilizumab (a specific antibody against the IL-6 receptor), but improved liver damage in mice with methionine choline-deficient (MCD) diet-induced NASH [23].

In NAFLD, negative regulation in the activity of the transcription factor peroxisome proliferator-activated receptor-*α* (PPAR-*α*) that reduces fatty acid (FA) oxidation, with a concurrent positive control in the activity of the lipogenic transcription factor sterol regulatory element-binding protein-1c (SREBP-1c), is reported [24]. These disturbances may enhance the ratio of hepatic *de novo* lipogenesis to fatty acid oxidation, with concomitant inhibition of FA export from the liver to other organs. Also, the reduction in activated PPAR-*α* levels may play a role in enhancing the DNA binding capacity of the proinflammatory transcription factors nuclear factor kappa B (NF-*κ*B) and activator protein 1 (AP-1), which represent the most important mechanisms for the progression of steatosis to NASH. These molecular changes favor a prolipogenic and proinflammatory liver state [24].

At present, there is a lack of consensus on the management of NAFLD, and, consequently, no drug is currently indicated for the treatment of NAFLD. However, since NAFLD is a multifactorial disease, approaches that combine reducing visceral adiposity, IR, and hyperinsulinemia, among others, have been indicated as a possible way out. Lifestyle intervention (diet, exercise) represents the mainstay of treatment [25].

Ruzu herbal bitters (RHB) is an aqueous preparation of *Curculigo pilosa* root (40%), *Uvaria chamae* stem (20%), and *Citrullus colocynthis* bark (40%) prepared in Nigeria. It has unverified claims for the management of arthritis, obesity, hypertension, infertility, diabetes, liver toning capacity, and arthritis among others. *C. pilosa* is used traditionally to treat heart diseases, impotence, gastrointestinal diseases, and arthritis [26]. A thorough examination of the phytochemical screening of *C. pilosa* shows that it possesses antioxidant, neuroprotective, and hepatoprotective activity [26]. A study reported the use of *U. chamae* for managing diarrhoea, cough, complicated abdominal pains, urinary tract, sickle cell anaemia, and cerebral infections and for preventing hepatic injuries [27]. The wide range of medicinal applications and uses of *C. colocynthis* plant is extensively acknowledged and reported [28]. The saponin extract of *C. colocynthis* fruits at several doses reduced the level of fasting blood glucose (FBG) in alloxan-induced diabetic rabbits significantly [29]. The hepatoprotective, antioxidant, and antilipidemic activities of RHB have been attributed to the presence of *U. chamae* and *C. colocynthis* [30].

RHB was recently demonstrated to have an ameliorative effect on antioxidant and biochemical abnormalities induced by a HFD in Wistar rats; its antilipidemic and antiobesity activities were also reported [30]. It is a polygenic mixture of high quantities of saponins, alkaloids, flavonoids, and cardiac glycosides [31]. However, its anti-inflammatory activity on NAFLD-induced HFD has not been studied. Hence, this study aimed to evaluate the anti-inflammatory activity of RHB in an experimental NAFLD animal model caused by a HFD.

## 2. Materials and Methods

### 2.1. Chemicals and Reagents

Chemicals used in this study include thiobarbituric acid (TBA), 4-(2-hydroxyethyl)piperazine-1-ethanesulfonic acid (HEPES), 1-chloro-2,4-dinitrobenzene (CDNB), ethylenediaminetetraacetic acid (EDTA), 5,5′-dithiobis(2-nitrobenzoic acid) (DTNB), pyrogallol, trichloroacetic acid (TCA), sodium hydroxide, hydrochloric acid, reduced glutathione (GSH).

Pioglitazone hydrochloride (>99% purity) was purchased from Tokyo Chemical Industry (Shanghai) Development Co. Ltd. Ruzu herbal bitters (RHB) was obtained from Seban Ventures, Ota, Ogun State, with NAFDAC Registration Number A7-1102L. Fenofibrate and cholesterol were also obtained from Fisher Scientific. All other reagents and chemicals used in the study were of analytical grade.

### 2.2. Experimental Animals

Male albino rats (*n* = 25) of Wistar strain weighing between 130 and 170 g were housed in propylene cages, kept under standardized laboratory settings, and provided free access to a standard and HFD and drinking water *ad libitum*. Rats were procured from Lagos State University Teaching Hospital, Idi Araba, Lagos State, Nigeria. The Institutional Animal Ethics Committee, Covenant University, approved all experiments and protocols described in the present study. The tests were performed in accordance with the “Guide for the Care and Use of Laboratory Animals” and “Committee for the Purpose of Control and Supervision of Experiments on Animals” (CPCSEA).

## 3. Experimental Diet

Diets used in this study were of two types, the standard rat chow and HFD, which were compounded (Tables 1 and 2) as reported by [2, 17] and manufactured by Graceline Feeds Ltd., Ota, Ogun State. The formulations are as described in Tables 1 and 2.

### 3.1. Experimental Design

Rats were acclimatized for two (2) weeks before the beginning of the experiment. The animals were weighed beforehand and weekly throughout the study duration. Thereafter, the rats were randomly divided into five groups with five rats in each group. Experimental design and treatments were as follows:

  Group 1, normal control: this group was fed on a standard chow diet and given normal saline (1 ml/kg body weight (BW)/day) by gastric intubation for twelve weeks.
  Group 2, pioglitazone (PIO) group: this group was fed on a HFD and given pioglitazone (4 mg/kg BW/day) by gastric intubation for twelve weeks.
  Group 3, Ruzu herbal bitters (RUZU) group: this group was fed on a HFD and given RUZU (0.6 ml/kg BW/day, an equivalent of the prescribed adult human dosage) by gastric intubation for twelve weeks.
  Group 4, negative control (NAFLD) group: this group was fed on a HFD and given normal saline (1 ml/kg BW/day) by gastric intubation for twelve weeks.
  Group 5, fenofibrate (FENO) group: this group was fed on a HFD and given fenofibrate (10 mg/kg BW/day) by subcutaneous injection for twelve weeks.

Eating pattern, body weights, and signs of abnormalities of the rats throughout the twelve-week experimental period were observed and recorded. At the end of the twelve weeks, a standard analytical kit (ACCU-CHECK Diagnostics, England) was used to carry out FBG evaluation. After that, the animals were sacrificed using mild anaesthesia (sodium pentobarbital), and blood was collected from the heart using heparinized syringes. The liver was rapidly dissected and washed free of blood with an ice-cold 0.9% NaCl solution. The weight of the liver was estimated and liver index calculated (liver weight/body weight × 100).

### 3.2. Sample Preparation

Whole blood centrifugation at 10,000 rpm for 5 min was carried out. Portions of each organ (brain, heart, kidney, liver, testes, lungs, spleen, pancreas, and intestine) were all collected after rinsing with normal saline to eliminate blood contamination and dried by blotting. These organs were homogenized in ice-cold homogenization buffer (0.25 M sucrose, 10 mM Tris-HCl, 1 mM EDTA, and 10 mM HEPES-NaOH at pH 7.4) using a Teflon pestle homogenizer. The homogenate was centrifuged at 12,000*g* for 30 minutes at 4°C temperature. The supernatant was collected and frozen at 20°C for enzymatic assays [30, 32]. A sizeable portion of the liver was stored in formalin for histology. The remaining tissues were then kept at −20°C until analysis. Liver markers of toxicity were evaluated in the plasma of experimental animals, while lipid peroxidation and antioxidant assessment were carried out in all excised organs.

### 3.3. Assessment of Antioxidant and Oxidative Stress Indices

Reduced glutathione (GSH) was assayed according to the method described in [33]. Lipid peroxidation was determined as malondialdehyde (MDA) level according to the method described by [34]. Superoxide dismutase (SOD) activities were determined according to [35].

### 3.4. Inflammatory Parameters

Plasma concentrations of TNF-*α*, IL-2, and IL-6 were quantified by the use of specific enzyme-linked immunosorbent assay (ELISA) kits procured from Proteintech Group Inc., USA.

### 3.5. Histological Examination

Liver tissues histology was carried out according to the method described by [36]. Briefly, the tissues were fixed, paraffin-embedded in paraffin wax, mounted on slides, deparaffinized, and stained using haematoxylin and eosin (H&E). The marked segments were observed under the microscope, and the images were captured.

### 3.6. Statistical Analysis

Data were considered using statistical package for the social sciences (SPSS version 23). The analyzed data were presented as mean ± SEM of five replicates in each group. Analysis of variance (ANOVA) was carried out to test for the level of homogeneity at *p* < 0.05 among the groups. Duncan's Multiple Range Test (DMRT) was used to separate the heterogeneous group.

## 4. Results

### 4.1. Effects on Body Weight

The body weights of the animals increased during the experiment (controls and treated). Body weights were estimated at the start and every week. By the 6^th^ week, the body weight in NAFLD rats started increasing significantly (*p* < 0.05) in comparison to PIO, RUZU, normal, and FENO groups (Table 3).

### 4.2. Effects on Liver Index

Table 4 shows the liver index percentage as compared with the control. There was a significant (*p* < 0.05) increase in the relative liver index of all the groups in comparison with control.

### 4.3. Effects on Oxidative Stress Markers

Oxidative stress markers of the liver of all the groups were examined.  Table 5 shows the concentration of malondialdehyde (MDA), reduced glutathione concentration (GSH), and activity of superoxide dismutase (SOD) in the liver. There was a significant increase in the level of MDA in the rats treated with NAFLD when compared with the control, but this was reversed with a significant reduction in the concentration of MDA in rats treated with RUZU when associated with the control. However, rats administered PIO and FENO also showed a significant decrease in this oxidative stress marker when compared to the NAFLD group. There was a significant reduction in the concentration of GSH and activity of SOD in rats administered NAFLD as compared to the control rats, but this was significantly increased in rats treated with RUZU when compared to the control rats. Rats administered PIO and FENO showed a significant increase in the antioxidant enzyme and molecule also.

### 4.4. Effects on Proinflammatory Cytokines

**Figure 1:**
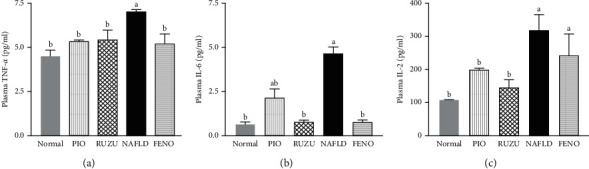
Effect on inflammatory cytokines (IL-2, IL-6, and TNF-*α*) in the liver. Values are presented as mean ± SEM (*n* = 5). ^a^*p* < 0.05, significant difference compared to normal control. ^b^*p* < 0.05, significant difference compared to NAFLD group. NAFLD: nonalcoholic fatty liver disease, PIO: pioglitazone (4 mg/kg body weight), RUZU: Ruzu herbal bitters (0.6 mg/kg body weight), and FENO: fenofibrate (10 mg/kg body weight).

There was a significant increase in concentrations of proinflammatory cytokines IL-6, IL-2, and TNF-*α* in the livers of rats administered HFD as compared to the control, but rats treated with RUZU after HFD were able to reverse this effect via a significant reduction in the proinflammatory cytokines when compared to the control group as shown in Figure 1. Furthermore, a significant increase in the liver index was observed in the NAFLD group when compared to the normal control.

### 4.5. Effects on Histological Studies

**Figure 2:**
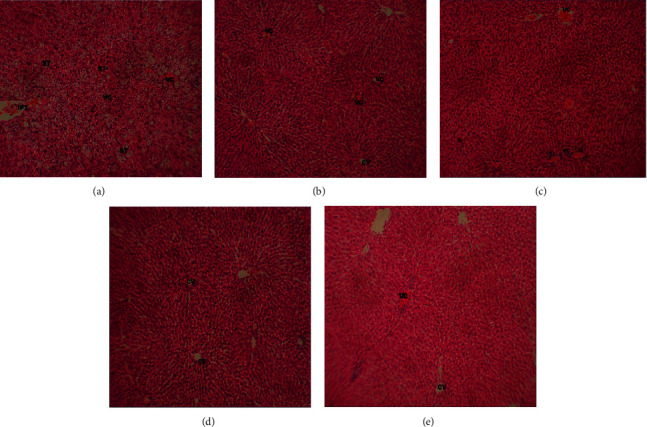
Histological changes in different groups. VC: vascular congestion, CV: central vein, IPT: inflammatory portal tract, and ST: steatosis (magnification: ×100). (a) Control group. (b) Pioglitazone (4 mg/kg body weight). (c) Ruzu herbal bitters (0.6 mg/kg body weight). (d) NAFLD. (e) Fenofibrate (10 mg/kg body weight).

Figures 2(a)–2(e) showed sections of liver histology from different groups of rats. The NAFLD group shows abnormal histology of the liver with a high level of steatosis (ST), vascular congestion (VC), and inflammatory portal tract (IPT). Aggregates of inflammatory cells are seen in the liver of the animals of NAFLD group compared to normal control. These observed alterations were ameliorated in animals fed HFD and treated with RHB, pioglitazone, and fenofibrate. The animals in these treated groups showed mild vascular congestion. The histology results of the animals treated with RHB reveal its hepatoprotective role when compared to the NAFLD animals.

## 5. Discussion

Over 80% of the world's population uses medicinal plants in the treatment of various forms of diseases. Statistically, the rate is higher in Africa, most especially in Nigeria [30]. NAFLD as a complex liver condition is documented as the hepatic indicator of metabolic syndrome, which spans a large spectrum of clinical-pathological conditions of the liver, ranging from steatosis to the end-stage liver cirrhosis [17]. In this study, twelve-week feeding of rats with HFD induced severe vascular congestion, steatosis, and a portal tract inflammation consistent with NAFLD. It also showed a typical histopathological lesion of NAFLD. This report is in line with that by Zaitone et al. [2]. The livers of rats fed a HFD for twelve or six weeks presented with modest to severe steatosis and lobular inflammation and developed typical histopathologic nonalcoholic steatohepatitis lesions [2]. The treatment of rats with RHB, pioglitazone, and fenofibrate ameliorated the histopathological effects to mild vascular congestion. This study shows that HFD induced a significant increase in the body weight of animals from the sixth week up to the twelfth week. The weight of the liver also increased significantly. This increase in weight is concordant with previous reports [17, 30]. The increase in weight is in tandem with the proposed pathogenesis for NAFLD, which has to do with increased fat aggregation.

Oxidative stress and inflammation play a major role in the pathogenesis of NAFLD [2]. Oxidative stress is a form of imbalance between oxidizing and reducing states of the body, resulting in the production and release of reactive species, which could invariably overwhelm the antioxidant defence systems of the body [37]. Oxidative stress and inflammation are known to be coconspirators. Therefore, induction of constant oxidative stress state could lead to an upsurge of inflammatory processes in the body. Thus, a remarkable increase in the liver MDA of NAFLD rats was detected when related to the control group. This significant increase in MDA shows that NAFLD induction could lead to the initiation of oxidative stress, which in turn may lead to severe liver injuries. This finding is consistent with earlier reports [2, 17, 30]. However, the administration of RHB, pioglitazone, and fenofibrate was able to reverse this effect, as there was a substantial decrease in the level of liver MDA. The observed significant reduction in the level and activity of liver GSH and SOD of the NAFLD group was in agreement with previous reports [2, 30, 37, 38]. This observed aberration in GSH content and SOD activities was, however, improved in the RHB administered groups and other positive controls. Increased oxidative stress has been related to dyslipidemia changes in obesity and, consequently, NAFLD, as there is inadequate capacity for the nonadipose tissues to preserve excess fats, and this fat build-up could trigger lipotoxicity and hence cell death [30].

Obesity and its related metabolic pathologies such as NAFLD are harmful metabolic diseases linked with a chronic inflammatory response categorized by increased acute-phase reactants, abnormal cytokine release, and activation of inflammatory signaling pathways [2]. The inflammatory activity of TNF-*α* is mainly employed by its ability to stimulate NF-kB, which results in the expression of inflammatory genes such as inflammatory cytokines, cell adhesion molecules, lipoxygenase, and cyclooxygenase-2. The modulation of cytokines linked with NAFLD and its connected inflammation has become a new focus of research. Circulating levels of TNF-*α*, IL-6, and others in NAFLD have been reported in literature [2, 38–40].

Furthermore, the upsurge in the concentrations of plasma TNF-*α*, IL-2, and IL-6, which are proinflammatory cytokines that are stimulated by lipid accumulation in the liver as a result of macrophage recruitment, was the catalyst for the progression of NAFLD [41]. However, the administration of RHB was, for the first time, reported in this study to favorably reduce TNF-*α*, IL-6, and IL-2. This reduction in the concentration of TNF-*α* was similarly reported [42]. The therapeutic options targeting the regulation of TNF-*α* have been proposed as a possible rational treatment for NAFLD. Consequently, this study shows that RHB was able to regulate the levels of examined cytokines, thereby reducing inflammation via the possible inactivation of NF-kB pathway and positive regulation of PPAR-*α* with the concurrent promotion of FA export from the liver to other organs, hence reducing the effect of NAFLD.

Further work in this study will involve the need to understand the molecular dynamics in favor of prolipogenic and proinflammatory liver conditions and the ameliorative roles of Ruzu herbal bitters in them.

## 6. Conclusion

The results of this study, for the first time, show that Ruzu herbal bitters (RHB) possesses hepatoprotective, anti-inflammatory, and antioxidant abilities in NAFLD-induced rats, and it might be considered as a suitable alternative therapy to pioglitazone and fenofibrate in the clinical management of hepatic disorders.

## Figures and Tables

**Table 1:** Diet composition in g/100 g.

	Normal diet	High-fat diet (HFD)
Maize	45	36.7
Flour binder	15	15
Soybeans	7	7
Groundnut cake	10	10
Fish	9	9
Oil	5	—
Corn offal	5	1
Bone	1.4	1.4
Vitamin premix	2	2
Lysine	0.1	0.1
Salt	0.2	0.2
Methionine	0.3	0.3
Lard	—	15.05
Cholesterol	—	2
Bile salt	—	0.25

**Table 2:** Premix contents.

Nutrients	Contents per 2.5 kg
Vitamin A	8,000,000 IU
Vitamin D3	1,500,000 IU
Vitamin E	7,000 mg
Vitamin K3	1,500 mg
Vitamin B1	2,000 mg
Vitamin B2	2,500 mg
Niacin	16,000 mg
Pantothenic acid	5,500 mg
Vitamin B6	2,000 mg
Vitamin B12	10 mg
Folic acid	500 mg
Biotin H2	250 mg
Chlorine chloride	175,000 mg
Manganese	40,000 mg
Selenium	200 mg
Antioxidant	3,750 mg

**Table 3:** The trend in body weight (grams) changes during the treatment period of 12 weeks.

Groups	Normal	PIO	RUZU	NAFLD	FENO
Start	170.00 ± 8.94	186.80 ± 7.50	180.80 ± 7.96	182.80 ± 9.34	196.00 ± 11.24
Week 2	182.60 ± 10.34	200.00 ± 6.54	195.60 ± 10.83	200.60 ± 5.02	205.20 ± 12.66
Week 4	196.80 ± 9.65	212.80 ± 9.65	206.40 ± 11.03	221.00 ± 5.60	214.40 ± 13.61
Week 6	205.20 ± 10.29^b^	222.00 ± 7.85^b^	218.00 ± 10.90^b^	256.20 ± 5.68^a^	226.00 ± 14.18^b^
Week 8	218.80 ± 10.59^b^	236.80 ± 6.86^b^	229.20 ± 11.77^b^	288.60 ± 6.19^a^	239.20 ± 15.60^b^
Week 10	231.20 ± 10.76^b^	251.60 ± 5.88^b^	238.80 ± 12.09^b^	310.80 ± 6.17^a^	250.80 ± 16.55^b^
Week 12	234.20 ± 9.47^b^	259.60 ± 4.78^b^	253.40 ± 14.65^b^	332.40 ± 7.23^a^	257.00 ± 15.82^b^

Values are presented as mean ± SEM (*n* = 5). ^a^*p* < 0.05, a significant difference compared to the normal control. ^b^*p* < 0.05, a significant difference compared to the NAFLD group. NAFLD: nonalcoholic fatty liver disease, PIO: pioglitazone (4 mg/kg body weight), RUZU: Ruzu herbal bitters (0.6 mg/kg body weight), and FENO: fenofibrate (10 mg/kg body weight).

**Table 4:** Effects of pioglitazone, RUZU, and fenofibrate on the liver index in the experimental groups.

Groups	Normal	PIO	RUZU	NAFLD	FENO
Liver index (%)	3.07 ± 0.03^b^	4.58 ± 0.05^ab^	3.93 ± 0.04^ab^	6.55 ± 0.30^a^	4.94 ± 0.06^ab^

Values are presented as mean ± SEM (*n* = 5).^a^*p* < 0.05, a significant difference compared to normal control. ^b^*p* < 0.05, a significant difference compared to the NAFLD group. NAFLD: nonalcoholic fatty liver disease, PIO: pioglitazone (4 mg/kg body weight), RUZU: Ruzu herbal bitters (0.6 mg/kg body weight), and FENO: fenofibrate (10 mg/kg body weight).

**Table 5:** Effects on oxidative stress markers in the liver tissue.

Groups	Normal	PIO	RUZU	NAFLD	FENO
MDA (*μ* moles/mg protein)	0.17 ± 0.01^b^	0.18 ± 0.02^b^	0.19 ± 0.02^b^	0.30 ± 0.01^a^	0.22 ± 0.02^b^
GSH (*n* moles/mg protein)	112.18 ± 6.18^b^	112.10 ± 3.79^b^	119.19 ± 9.21^b^	72.24 ± 5.15^a^	151.53 ± 19.69^ab^
SOD (units/mg protein)	0.86 ± 0.07^b^	1.09 ± 0.14^b^	0.79 ± 0.03^b^	0.47 ± 0.07^a^	0.76 ± 0.18^b^

Values are presented as mean ± SEM (*n* = 5). ^a^*p* < 0.05, a significant difference compared to the normal control. ^b^*p* < 0.05, a significant difference compared to the NAFLD group. NAFLD: nonalcoholic fatty liver disease, PIO: pioglitazone (4 mg/kg body weight), RUZU: Ruzu herbal bitters (0.6 mg/kg body weight), and FENO: fenofibrate (10 mg/kg body weight).

## Data Availability

The experimental data used to support the findings of this study are included within the article.
